# Associations between Longitudinal Gestational Weight Gain and Scalar Infant Birth Weight: A Bayesian Joint Modeling Approach

**DOI:** 10.3390/e24020232

**Published:** 2022-02-02

**Authors:** Matthew Pietrosanu, Linglong Kong, Yan Yuan, Rhonda C. Bell, Nicole Letourneau, Bei Jiang

**Affiliations:** 1 Department of Mathematical and Statistical Sciences, University of Alberta, Edmonton, AB T6G 2G1, Canada; pietrosa@ualberta.ca (M.P.); lkong@ualberta.ca (L.K.); 2 School of Public Health, University of Alberta, Edmonton, AB T6G 1C9, Canada; yyuan@ualberta.ca; 3 Department of Agricultural, Food & Nutritional Science, University of Alberta, Edmonton, AB T6G 2P5, Canada; bellr@ualberta.ca; 4 Faculty of Nursing and Cumming School of Medicine, University of Calgary, Calgary, AB T2N 1N4, Canada; nicole.letourneau@ucalgary.ca

**Keywords:** Bayesian modeling, functional regression, gestational weight, infant birth weight, joint modeling, longitudinal data, maternal weight gain

## Abstract

Despite the importance of maternal gestational weight gain, it is not yet conclusively understood how weight gain during different stages of pregnancy influences health outcomes for either mother or child. We partially attribute this to differences in and the validity of statistical methods for the analysis of longitudinal and scalar outcome data. In this paper, we propose a Bayesian joint regression model that estimates and uses trajectory parameters as predictors of a scalar response. Our model remedies notable issues with traditional linear regression approaches found in the clinical literature. In particular, our methodology accommodates nonprospective designs by correcting for bias in self-reported prestudy measures; truly accommodates sparse longitudinal observations and short-term variation without data aggregation or precomputation; and is more robust to the choice of model changepoints. We demonstrate these advantages through a real-world application to the Alberta Pregnancy Outcomes and Nutrition (APrON) dataset and a comparison to a linear regression approach from the clinical literature. Our methods extend naturally to other maternal and infant outcomes as well as to areas of research that employ similarly structured data.

## 1. Introduction

Maternal weight gain supports fetal growth and holds important health implications for both mother and child during and after pregnancy [1,2,3]. Insufficient weight gain is associated with preterm birth and low infant birth weight, while excessive weight gain is linked to postpartum weight retention, gestational diabetes, hypertension, infant macrosomia, and other complications [3,4,5]. A growing amount of clinical literature further implicates maternal gestational weight gain outside of recommendations in adverse, long-term health outcomes for the child, including a heightened future risk of cardiovascular disease [6,7].

It is not yet conclusively understood how weight gain in different stages of pregnancy affects health outcomes for either mother or child. This is despite previous findings that gestational weight trajectories are similar across human populations with varying genetic, cultural, and lifestyle traits [8]. As an example central to this article, previous studies present conflicting conclusions on the effect of first- and second-trimester weight gain on infant birth weight [8,9,10,11,12]. We attribute this in part to differences in and the validity of the statistical methods currently used to jointly analyze scalar outcomes and longitudinal data. Thus, developments in methodology for analyzing how patterns in longitudinal data (e.g., gestational weight gain) influence scalar outcomes (e.g., infant birth weight) are both statistically and clinically relevant.

Retnakaran et al. [13] investigates the relationship between infant birth weight and gestational weight gain in different periods of pregnancy using traditional linear regression. The work’s models include, as predictors, demographic covariates together with pregravid weight and interval-specific average weight gain. The authors opt for clinical data in order to avoid bias in self-reported pregravid measurements that they claim is prevalent in other studies [5,12]. The resulting preconception study design presents a few practical problems: this design is more difficult to implement, limits the use of secondary data, and can introduce other sampling biases and restrict model generalizability (e.g., through the exclusion of unplanned pregnancies). Despite the supposed benefit of bias reduction, the work’s average weight gain measurements are precomputed (as differences in average weight between gestational intervals) and may be highly variable due to clinical measurement error and the small number of observations in each gestational interval. As Richardson notes, ignoring this measurement error can lead to unreliable effect estimates and misleading conclusions [14]. This linear regression approach furthermore does not account for gestational age at each weight measurement and, through its initial precomputing stage, reduces the amount of data used to fit the model. The consequent coarsening of information may contribute to unreliable effect estimates and conclusions.

To address these issues, we turn to other approaches for modeling longitudinal data. Joint models that simultaneously consider longitudinal responses and scalar health outcomes are well established in the statistical literature [15,16,17,18,19,20,21,22]. These models were originally motivated by HIV/AIDS and cancer research to predict patient outcomes using a time-dependent covariate trajectory. Relevant methodology has since evolved to incorporate techniques from functional data analysis, semiparametric inference, robust estimation, and Bayesian methods [23].

In this paper, we consider a joint model for infant birth weight and gestational weight gain trajectories that also incorporates clinical covariates. Our approach efficiently uses information from estimated mean weight trajectories—including estimated pregravid weight, interval-specific rates of weight gain, and individual residual variance—to predict infant birth weight. As a result, our model can correct for bias in self-reported weight measurements (when combined with clinical observations) and permits nonprospective study designs with unbalanced longitudinal observations.

We employ the Bayesian joint modeling approach of Jiang et al. [23]. Our model uses parameter estimates that describe individual gestational weight trajectories to model the association between infant birth weight and gestational weight gain. We model the mean [24,25] and measurement error [26,27] of these trajectories using a robust, semiparametric mixed effects model and a Bayesian linear spline approach [23].

Our joint model remedies the issues noted above for linear regression [13]. First, by using estimated mean trajectory parameters as predictors of infant birth weight, our approach obtains more-efficient estimates of the time-dependent effects of gestational weight gain. More generally, our joint modeling method, implemented in a Bayesian framework, borrows information from all observations and patients in a one-stage procedure. On the other hand, the predictors in the traditional linear model, such as interval-specific weight gain, are precomputed in an initial step independently for each patient using only a small proportion of the available data at a time. Second, our approach truly accommodates longitudinal data by explicitly accounting for gestational age at each weight measurement when estimating weight gain trajectories. Third, unlike other studies that treat within-patient residual variance as a nuisance parameter, our method models measurement error variance and uses it as a random effect to predict infant birth weight.

Our approach to mean trajectory modeling mitigates bias in self-reported prestudy measurements and accounts for variability inherent in observed data. These are notable advantages over traditional methods such as the linear regression approach above, where the amalgamation of data from different sources can negatively impact an analysis. Another advantage of the proposed model is its potential to be used for prediction and intervention: our model can be applied to predict infant birth weight well before term and can thus be conveniently deployed in clinical settings. More generally, while infant birth weight is the primary focus of the present paper, our approach and discussions apply to other maternal and infant outcomes and to other areas of research that employ similarly structured data.

In Section 2, we introduce the pregnancy outcomes dataset used in this article and the proposed model. This section also presents our chosen prior distributions and computational methods. We present estimates for the effect of time-specific maternal weight gain on infant birth weight obtained under the proposed model in Section 3, and compare these estimates to those obtained using the linear regression approach described above [13]. In Section 4, we discuss our results and provide some concluding remarks on the general significance of our approach and future directions.

## 2. Materials and Methods

### 2.1. Data

Throughout this paper, we use data from the 2009–2012 Alberta Pregnancy Outcomes and Nutrition (APrON) study [28]. The 2189 women in the APrON study, all of whom were at least 16 years of age and at most 27 weeks into gestation, are part of a longitudinal cohort [28,29]. As part of the APrON study, maternal weight and gestational age were measured at each trimester following registration. Participants recruited before 13 weeks gestation have measurements corresponding to all three trimesters, while those recruited between 14 and 27 weeks gestation have measurements only for the second and third trimesters. Pregravid weight, along with other demographic characteristics, were self-reported by each participant upon recruitment. Gestational age at delivery was assessed postpartum. In addition to the APrON data, clinical weight measurements were collected from all participants at regularly scheduled prenatal visits. The number of weight measurements for each participant varies due to missing appointments or data. The longitudinal weight data in this study may be considered sparse and has been previously examined in the functional data analysis literature [30].

We only include participants with a live, singleton birth in the following analyses. We exclude individuals without a reported pregravid weight; those with less than three weight measurements during pregnancy; and those with missing gestational age at delivery, infant birth weight, marital status, education level, income level, ethnic origin, parity, or age. We do not consider any postpartum weight measurements in our analyses.

The final analytic sample consists of *n* = 1340 participants with *N* = 15,183 weight observations. Demographic characteristics for this sample, stratified by infant birth weight class, are summarized in Table 1. We use <2.5 kg, ≥2.5 kg and <4 kg, and ≥4 kg as criteria defining low, normal, and high infant birth weight classes [31]. Clinical weight measurements (i.e., not including self-reported pregravid measurements) were taken at gestational ages ranging from 4.4 to 41.7 weeks, with a median of 30.3 weeks. Participants have a median of 12 recorded weight measurements each.

### 2.2. Joint Model

We now present our joint model for infant birth weight and longitudinal gestational weight gain. As a main feature, the model estimates the former using parameter estimates from patient-specific maternal weight trajectories:

$$\begin{array}{cc} Y_{i}|b_{i} & =\left(1,z_{i}^{⊤},b_{i}^{⊤},\ln\sigma_{i}^{2}\right)\theta +\varepsilon_{i}\\ \varepsilon_{i} & \overset{i.i.d.}{∼}N\left(0,\sigma^{2}\right) \end{array}$$

for i=1,⋯,n, where $Y_{i}$ denotes an observed infant birth weight; $z_{i}$ an observed demographic covariate vector; $b_{i}$ a vector of random weight trajectory parameters; and $\sigma_{i}^{2}$ the trajectory’s residual variance for the *i*th patient. The vector θ contains the corresponding fixed and random effects.

Individual longitudinal weight trajectories influence $Y_{i}$ through the random trajectory parameters $b_{i}$ in the longitudinal submodel

$$\begin{array}{cc} X_{ij} & =f\left(t_{ij};b_{i}\right)+\varepsilon_{ij}\\ \varepsilon_{ij} & \overset{i.i.d.}{∼}N\left(0,\sigma_{i}^{2}\right)\\ b_{i} & \overset{i.i.d.}{∼}N\left(\beta ,\Sigma \right) \end{array}$$

for $j=1,\cdots ,n_{i}$, where $X_{ij}$ is the observed weight of the *i*th patient at gestational age $t_{ij}$ and $n_{i}$ is the total number of longitudinal observations for the *i*th patient. We consider a piecewise linear weight trajectory (as a function of gestational age t≥0) [32]

$$f\left(t;b={\left(b_{0},b_{1},\cdots ,b_{K}\right)}^{⊤}\right)=b_{0}+\sum_{k=1}^{K}b_{k}{\left(t-t_{k}^{*}\right)}_{+},$$

where $x_{+}=\max\left\{0,x\right\}$ for x∈R and $\left(t_{1}^{*}=0,\cdots .t_{K}^{*},t_{K+1}^{*}=\infty \right)$ is a fixed, increasing sequence of changepoint locations. Consequently, $b_{0}$ is the mean pregravid weight and $\sum_{k=1}^{k_{0}}b_{k}$ is the mean rate of weight gain in the gestational age interval $\left[t_{k_{0}}^{*},t_{k_{0}+1}^{*}\right)$, for $k_{0}=1,\cdots ,K$. Following common trimester boundaries [13], we take K=8 with $t_{2}^{*}=13$, $t_{3}^{*}=18$, $t_{4}^{*}=23$, $t_{5}^{*}=27$, $t_{6}^{*}=32$, $t_{7}^{*}=37$, and $t_{8}^{*}=45$.

Under the proposed model, β describes an average, “prototype” trajectory, while the random $b_{i}$s describe patient-specific trajectories and deviations from β. Our longitudinal model accounts for short-term variation and measurement error in patient trajectories by using $\ln\sigma_{i}^{2}$ as a predictor of $Y_{i}$.

### 2.3. Bayesian Framework and Model Estimation

We take a Bayesian approach to parameter estimation in the proposed model.

In the longitudinal submodel, we model random trajectory parameters as $b_{i}\overset{i.i.d.}{∼}N\left(\beta ,\Sigma \right)$ under the diffuse prior β∼N(0,10I). Additional tests, not presented here, indicate no need to consider a Gaussian mixture [23] in the distribution of the $b_{i}$s for our APrON dataset. To avoid issues with unbounded likelihood [33] when using an unstructured random effect covariance matrix Σ, we implement the empirical Bayes Wishart prior [34]

$$\Sigma ∼W\left(m=2+\frac{K+1}{2},\;\;\Lambda =\sum_{i=1}^{n}\hat{\mathrm{Cov}}({\hat{b}}_{i}^{\left(\mathrm{OLS}\right)}{)}^{-1}\right),$$

where $\hat{\mathrm{Cov}}({\hat{b}}_{i}^{\left(\mathrm{OLS}\right)})$ is an estimate of the covariance matrix of the ordinary least squares (OLS) estimator of $b_{i}$. For the $\sigma_{i}^{2}$s, the trajectory residual variances, we assume a log-normal prior $\ln\sigma_{i}^{2}\overset{i.i.d.}{∼}N\left(\mu ,\tau^{2}\right)$ under the diffuse hyperpriors $\mu ∼N(0,10^{3})$ and $\tau^{2}∼\mathrm{Inv}-\mathrm{Gamma}\left(10^{-4},10^{-4}\right)$. For the scalar response $Y_{i}$, we take θ∼N(0,10I) and $\sigma^{2}∼\mathrm{Inv}-\mathrm{Gamma}\left(10^{-4},10^{-4}\right)$.

For notational simplicity, let $\varphi =\{\theta ,\sigma^{2},\beta ,\Sigma ,\mu ,\tau^{2}\}$ be the collection of model parameters. We assume that all elements of φ have independent prior distributions and denote the joint prior of φ by π. Define $\eta_{i}^{\mu }=\left(1,z_{i}^{⊤},b_{i}^{⊤},\ln\sigma_{i}^{2}\right)\theta$ as the linear predictor corresponding to $Y_{i}$.

The full likelihood of φ for our model is

$$\begin{array}{cc} L\left(\varphi \right)=\pi \left(\varphi \right)\prod_{i=1}^{n}[ & {\left|\Sigma \right|}^{-0.5}\exp\left\{-0.5{\left(b_{i}-\beta \right)}^{⊤}\Sigma^{-1}\left(b_{i}-\beta \right)\right\}\\ \; & \times \prod_{j=1}^{n_{i}}\left[\sigma_{i}^{-1}\exp\left\{-0.5\sigma_{i}^{-2}{\left(x_{ij}-f\left(t_{ij};b_{i}\right)\right)}^{2}\right\}\right]\\ \; & \times \tau^{-1}\exp\left\{-0.5\tau^{-2}{\left(\ln\sigma_{i}^{2}-\mu \right)}^{2}\right\}\\ \; & \times \sigma^{-1}\exp\left\{-0.5\sigma^{-2}{\left(y_{i}-\eta_{i}^{\mu }\right)}^{2}\right\}]. \end{array}$$

We implement a Gibbs sampler to perform posterior draws. For analytic derivations of the posterior distributions, see Jiang et al. [23]. As the full conditional posterior of $\sigma_{i}^{2}$ has no closed form, we obtain draws using the inverse cumulative distribution function method. In our Markov Chain Monte Carlo (MCMC) procedure, we run a chain of 150,000 iterations and use the first 50,000 iterations as a burn-in period; however, in this particular application, we observe that the model converges very quickly and that even 10,000 total iterations are sufficient. To reduce autocorrelation in subsequent draws, we thin posterior draws by saving only every 10th. We implement our model in C++ using the Scythe open-source statistical library [35] and R [36].

We consider two models, each accounting for a different set of demographic covariates. The first model (JM1) includes education level, income level, ethnic origin, parity, age at pregnancy, and gestational age at delivery. The second model (JM2) includes only demographic variables whose 95% credible interval in JM1 do not contain zero.

### 2.4. Comparison to Linear Regression

We compare our proposed method against the previously noted traditional linear regression (LR) approach. We focus specifically on differences in the effects of maternal weight gain rate in different gestational age periods on infant birth weight. To make this comparison easier, we use the rate of weight gain in each gestational period (rather than period-specific absolute weight gain) as a predictor of infant birth weight $Y_{i}$.

We use the same gestational age intervals in both models: [0,13), [13,18), [18,23), [23,27), [27,32), [32,37), and [32,45). To compute the average rate of weight gain ${\tilde{b}}_{k}$ in the *k*th interval, we first calculate the averages, $\mu_{k}$ and $\mu_{k-1}$, of weight measurements taken in the *k*th and (k−1)th intervals, respectively. We then calculate the rate of weight gain as ${\tilde{b}}_{k}=\left(\mu_{k}-\mu_{k-1}\right)/\left(m_{k}-m_{k-1}\right)$, where $m_{k}$ is the midpoint of the *k*th gestational age interval. For the sake of notation, we let k=0 refer to pregravid measurements (i.e., at week zero).

As noted previously, our joint model addresses numerous shortcomings of the LR approach. First, the LR model does not fully take into account the timing of individual maternal weight measurements, while our JM approach estimates patient-specific weight trajectories as functions of time. Second, LR model estimates are subject to short-term measurement error and variability: this is because only a small number of measurements contribute to pregravid weight and the estimated rates of weight gain. Our hierarchical Bayesian framework borrows information from all observations to estimate these quantities via patient-specific trajectory parameters. As another feature that may be clinically relevant in some applications, our model also estimates and uses short-term variability in maternal weight as another predictor.

We similarly consider two linear regression models in the following analyses. The first (LR1) uses estimated rates of weight gain (i.e., the ${\tilde{b}}_{k}$s), average pregravid weight ${\tilde{b}}_{0}=\mu_{0}$, and the same demographic variables as JM1. Similar to JM2, the second model (LR2) includes only the demographic covariates whose 95% confidence intervals in LR1 do not contain zero.

## 3. Results and Discussion

Table 2 presents parameter estimates for all four of the models described in the previous section. Model convergence for the joint models were assessed visually and numerically using five parallel chains. Trace plots for each of the coefficients in Table 2 suggest adequate convergence and mixing. Numerically, Rubin–Gelman statistics [37] for these coefficients range from 1.005 to 1.027 and also imply model convergence.

We observe major differences in the estimated effects of weight gain between the LR and JM approaches. Both LR models find rate of weight gain to be a useful predictor of infant birth weight only after 18 weeks gestation. On the other hand, the JM models find this to be true throughout gestation, including before 18 weeks.

Further, we note a difference in the direction of the estimated effect of weight gain during weeks 32–37 between the JM and LR models. Our JM approach estimates this effect to be positive, while the LM model estimates a negative effect. Given the positive estimates for other gestational intervals and the positive estimate originally reported in Retnakaran et al. [13], we suspect that the LR model is inaccurate here. As discussed previously, this could be attributed to the loss of time information or the precomputation of average weight gain measurements. These results illustrate how the LR approach might not yield reliable conclusions, even with relatively large datasets. Towards the end of this section, we also discuss the sensitivity of the LR approach to the choice of gestational intervals.

Other differences in the effect of rate of weight gain are less drastic but important nonetheless. In general, effect estimates in the LR models (relative to those in the JM models) are shrunk towards zero. We attribute this shrinkage to attenuation bias in the LR models due to self-reporting bias (in pregravid measurements) and the LR models’ inability to account for short-term variation in the weight trajectories. As discussed previously, this can be due to the small number of observations used to compute each patient’s pregravid weight (${\tilde{b}}_{0}$) and interval-specific rates of weight gain (the ${\tilde{b}}_{k}$s).

**Figure 1:**
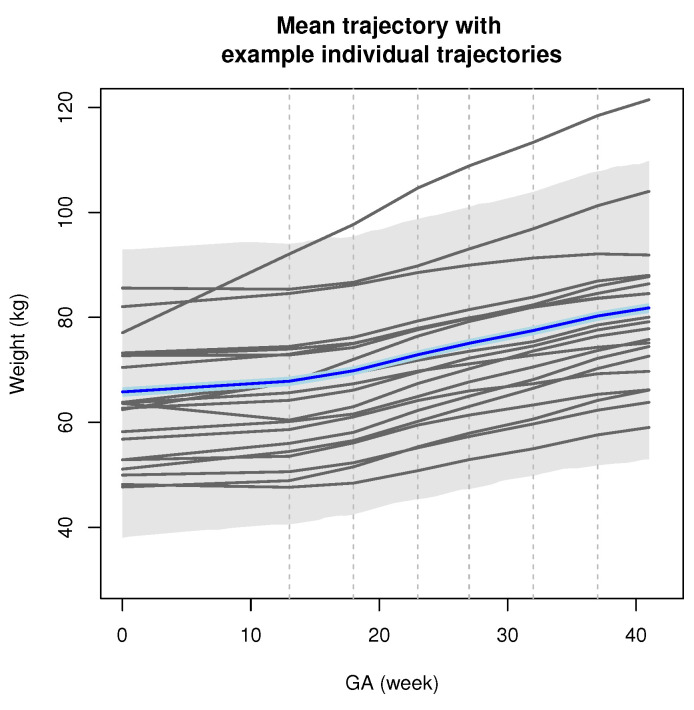
Posterior mean estimates from the proposed JM1 model for the mean weight gain trajectory β (solid blue) and twenty randomly selected individual trajectories $b_{i}$ (solid grey), both as functions of gestational age (GA). The light blue and grey regions describe 95% credible bands for β and $b_{i}$, respectively. Dotted grey lines indicate model changepoints (i.e., at $\mathrm{GA}=t_{k}^{*}$).

**Figure 2:**
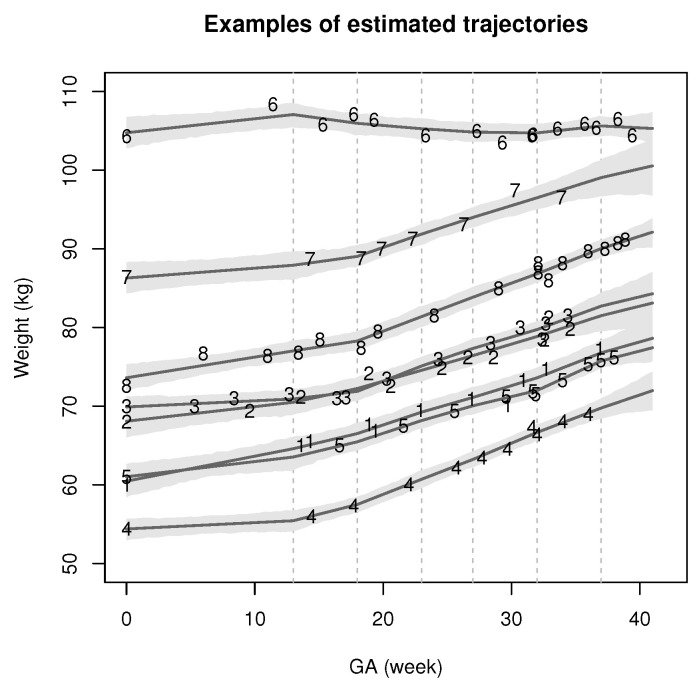
Eight randomly selected estimates of individual trajectories $b_{i}$ from the JM1 model as functions of gestational age (GA) (solid grey) and corresponding observed weights $X_{ij}$. Observed weights from the eight patients are denoted by 1,2,⋯,8. Light grey regions denote 95% credible bands for $X_{ij}$ (each for a fixed *i*). Dotted grey lines indicate model changepoints (i.e., at $\mathrm{GA}=t_{k}^{*}$).

Figure 1 illustrates the importance of accounting for deviation in patient-level trajectories (described by the $b_{i}$s) from the prototype trajectory (described by β) in our JM approach. While an overall trend in individual fitted trajectories is apparent, we see significant amounts of variation in gestational weight gain trajectories between patients. Figure 2 illustrates our proposed model’s ability to accommodate individual longitudinal trajectories even in the presence of between-patient variability.

In a separate analysis not shown in Table 2, we consider a different set of gestational intervals (i.e., the sequence of $t_{k}^{*}$s): [0,15), [15,20), [20,25), [25,30), [30,35), and [35,45), this time chosen out of convenience. The JM models yield similar conclusions with these different intervals while the LR models find weight gain during only 20–30 weeks gestation to be associated with infant birth weight. This demonstrates that the LR model is not robust with respect to the precomputation of interval-specific weight gain measurements and, as above, calls into question the validity of this approach.

## 4. Conclusions

In this paper, we provided a hierarchical Bayesian model for the joint analysis of scalar and longitudinal data based on Jiang et al. [23]. Our work was motivated by a question in maternal health research on the relationship between (scalar) infant birth weight and (longitudinal) gestational weight gain during different periods of pregnancy. We contrasted our joint modeling approach with one using traditional linear regression that has appeared in the clinical literature [13] and is reminiscent of analyses commonly seen in applied research.

This comparative LR approach was originally proposed for a preconception cohort study to eliminate self-reporting bias in pregravid measurements [13]. However, in addition to the design’s inconvenience, this approach does not fully account for gestational age or clinical measurement error and uses only a small number of observations to pre-estimate (i.e., in an initial stage separate from model estimation) weight gain in each gestational period. This results in high-variance model estimates that are not robust to the choice of gestational intervals. In contrast, through a one-stage, hierarchical Bayesian framework, our JM approach accounts for gestational age and short-term variability in longitudinal measurements, and borrows information from all observations to reduce bias and obtain more-reliable estimates.

The benefits of our model over the LR approach are apparent in our real-world study using the APrON pregnancy outcomes dataset. Beyond the LR model’s questionable negative estimated association between infant birth weight and maternal weight gain for 32–37 weeks gestation, we observed relative shrinkage in LR effect estimates towards zero. This illustrates the unreliability of the LR methodology and the impact of attenuation bias on effect estimates. On the other hand, our JM approach produced estimates that were reasonable and stable, even when considering different gestational periods.

We have demonstrated the usefulness of our joint modeling approach in settings with continuous scalar and longitudinal responses. Our approach extends naturally to other submodels and data types such as ordinal health outcomes (e.g., through an appropriate (cumulative) probit or logit link function at the response level of the model) [23]. While our focus in this paper was on comparing the JM and LR approaches, the proposed model can be further optimized for predictive purposes. Our developments hold immediate implications for clinical interventions, such as the early identification of pregnant women at risk of birth complications (e.g., extreme infant birth weight or other outcomes, whether scalar or ordinal) using self-reported prepregnancy data or sparse clinical observations.

## Figures and Tables

**Table 1:** Summary of demographic covariates for the analytic sample in the APrON dataset. For categorical variables, counts and relative percentages are reported. A * indicates the chosen reference category. For continuous variables, means (and standard deviations, in parentheses) are reported.

	Infant Birth Weight Class		
	Low (<2.5 kg)	Normal (≥2.5 and <4 kg)	High (≥4 kg)
**Mother characteristics**			
*Participants*	56 (4.18%)	1163 (86.79%)	121 (9.03%)
*Age, years*	32.66 (4.76)	31.33 (4.27)	31.91 (4.03)
*Marital status*			
Married *	55 (98.21%)	1122 (96.47%)	118 (97.52%)
Single	1 (1.79%)	41(3.53%)	3 (2.48%)
*Education*			
Graduate degree	11 (19.64%)	273 (23.47%)	27 (22.31%)
Some post-secondary *	37 (66.07%)	775 (66.64%)	83 (68.60%)
High school	8 (14.29%)	115 (9.89%)	11 (9.09%)
*Income level*			
<70 k	11 (19.64%)	234 (20.12%)	19 (15.70%)
≥70 k *	45 (80.36%)	929 (79.88%)	102 (84.30%)
*Ethnic origin*			
Asian	8 (14.29%)	77 (6.62%)	0 (0.00%)
Black	4 (7.14%)	11 (0.95%)	0 (0.00%)
Caucasian *	37 (66.07%)	956 (82.20%)	114 (94.22%)
Latin American	1 (1.79%)	38 (3.27%)	3 (2.48%)
Southeast Asian	4 (7.14%)	53 (4.56%)	2 (1.65%)
Other	2 (3.57%)	28 (2.41%)	2 (1.65%)
*Parity*			
0 *	35 (62.50%)	667 (57.35%)	48 (39.67%)
1	18 (32.14%)	387 (33.28%)	52 (42.98%)
≥2	3 (5.46%)	109 (9.37%)	21 (17.36%)
**Child characteristics**			
*Birth weight, kg*	2.23 (0.34)	3.33 (0.35)	4.25 (0.21)
*Gestational age at delivery, weeks*	36.01 (2.57)	39.51 (1.27)	40.20 (1.02)

**Table 2:** Parameter estimates obtained using the LR and the proposed JM models, with 95% confidence and credible intervals, respectively. For JM model interpretability, we present estimates for $\sum_{j=1}^{k}b_{j}$ (rather than for just $b_{k}$), which can be interpreted as the effect of weight gain rate in the *k*th gestational interval. Boldface indicates an estimate whose corresponding credible (or confidence) interval does not contain zero.

	Model			
	JM1	JM2	LR1	LR2
**Demographic variables**				
*Marital status*				
Single	0.151 (−0.161, 0.467)		**0.277** **(0.001, 0.553)**	
*Education*				
Graduate	0.016 (−0.115, 0.146)		0.059 (−0.058, 0.175)	
High school	−0.034 (−0.222, 0.152)		−0.114 (−0.340, 0.112)	
*Income level*				
<70 k	−0.039 (−0.193, 0.114)		−0.137 (−0.291, 0.016)	
*Ethnic origin*				
Asian	−0.038 (−0.264, 0.183)		−0.025 (−0.227, 0.177)	
Black	−0.322 (−0.856, 0.208)		0.056 (−0.805, 0.917)	
Latin American	−0.038 (−0.356, 0.272)		−0.144 (−0.419, 0.131)	
Southeast Asian	−0.098 (−0.369, 0.178)		−0.139 (−0.396, 0.118)	
Other	−0.09 (−0.445, 0.269)		**0.348** ** (0.027, 0.670)**	
*Parity*				
1	**0.147** ** (0.028, 0.269)**	**0.136** ** (0.020, 0.254)**	**0.137** ** (0.017, 0.258)**	**0.121** ** (0.005, 0.238)**
≥2	**0.246** ** (0.052, 0.444)**	**0.215** ** (0.032, 0.400)**	**0.384** ** (0.206, 0.561)**	**0.331** ** (0.159, 0.503)**
*Age at pregnancy*	−0.013 (−0.073, 0.047)		−0.025 (−0.082, 0.032)	
*Gestational age at delivery*	**0.162** ** (0.127, 0.197)**	**0.166** ** (0.130, 0.200)**	**0.092** ** (0.039, 0.145)**	**0.103** ** (0.051, 0.155)**
**Pre-pregnancy weight**				
*Clinical measure*			**0.006** ** (0.002, 0.011)**	**0.006** ** (0.002, 0.011)**
*Trajectory estimate (${\hat{b}}_{0}$)*	**0.007** **(0.003, 0.012)**	**0.007** **(0.003, 0.012)**		
*Trajectory estimator variance* ($\ln{\hat{\Sigma }}_{11}$)	**0.162** ** (0.127, 0.197)**	0.003 (−0.063, 0.066)		
**Rate of weight gain (by GA interval)**				
[0,13)	**0.701** ** (0.264, 1.138)**	**0.718** ** (0.300, 1.153)**	0.061 (−0.186, 0.307)	0.085 (−0.157, 0.327)
[13,18)	**1.256** ** (0.527, 1.972)**	**1.291** ** (0.581, 2.014)**	0.076 (−0.186, 0.333)	0.123 (−0.129, 0.375)
[28,23)	**1.703** ** (0.697, 2.708)**	**1.758** ** (0.780, 2.728)**	**0.201** ** (0.032, 0.371)**	**0.200** ** (0.034, 0.365)**
[23,27)	**1.929** ** (0.665, 3.183)**	**1.997** ** (0.754, 3.219)**	**0.191** ** (0.026, 0.356)**	**0.193** ** (0.031, 0.356)**
[27,32)	**2.010** ** (0.490, 3.525)**	**2.082** ** (0.613, 3.538)**	**0.270** ** (0.102, 0.437)**	**0.223** ** (0.06, 0.385)**
[32,37)	**2.009** ** (0.390, 3.673)**	**2.086** ** (0.455, 3.662)**	**−0.277** ** (−0.507, −0.048)**	**−0.285** ** (−0.513, −0.056)**
[37,45)	**2.027** ** (0.330, 3.720)**	**2.108** ** (0.439, 3.729)**	**0.277** ** (0.002, 0.551)**	**0.304** ** (0.033, 0.574)**

## Data Availability

Data are available from the Secondary Analyses to Generate Evidence (SAGE) databases held within the Policy Wise for Children and Families (nongovernmental) organization in Alberta, Canada: https://policywise.com/, accessed on 16 December 2021. Data are available subject to appropriate review and approvals. Access to Alberta Pregnancy Outcomes and Nutrition (APrON) data is administered by SAGE: requests can be made to data@policywise.com.
